# A Light Spot Humanoid Motion Paradigm Modulated by the Change of Brightness to Recognize the Stride Motion Frequency

**DOI:** 10.3389/fnhum.2018.00377

**Published:** 2018-10-15

**Authors:** Xin Zhang, Guanghua Xu, Xun Zhang, Qingqiang Wu

**Affiliations:** ^1^School of Mechanical Engineering, Xi’an Jiaotong University, Xi’an, China; ^2^State Key Laboratory for Manufacturing Systems Engineering, Xi’an Jiaotong University, Xi’an, China

**Keywords:** brain–computer interface, steady-state visual evoked potential, motion modulated by the change of brightness, same frequency stimulation, stride motion frequency

## Abstract

The steady-state visual evoked potential (SSVEP)-based brain–computer interface (BCI) usually has the advantages of high information transfer rate (ITR) and no need for training. However, low frequencies, such as the human stride motion frequency, cannot easily induce SSVEP. To solve this problem, a light spot humanoid motion paradigm modulated by the change of brightness was designed in this study. The characteristics of the brain response to the motion paradigm modulated by the change of brightness were analyzed for the first time. The results showed that the designed paradigm could induce not only the high flicker frequency but also the modulation frequencies between the change of brightness and the motion in the primary visual cortex. Thus, the stride motion frequency can be recognized through the modulation frequencies by using the designed paradigm. Also, in an online experiment, this paradigm was employed to control a lower limb robot to achieve same frequency stimulation, which meant that the visual stimulation frequency was the same as the motion frequency of the robot. Also, canonical correlation analysis (CCA) was used to distinguish three different stride motion frequencies. The average accuracies of the classification in three walking speeds using the designed paradigm with the same and different high frequencies reached 87 and 95% respectively. Furthermore, the angles of the knee joint of the robot were obtained to demonstrate the feasibility of the electroencephalograph (EEG)-driven robot with same stimulation.

## Introduction

The steady-state visual evoked potential (SSVEP) is an electrophysiological signal evoked by periodic visual stimulation, with a stationary distinctive spectrum showing characteristic SSVEP peaks, stable over time (Vialatte et al., 2010). It has the advantages of high information transfer rate (ITR) and no need for training (Chen et al., 2015). Thus, it has attracted more and more attention. Since the cones cells concentrate at the center of the retina (McFarland and Wolpaw, 2011), the eye’s sensitivity to visual stimuli is the highest at the center of the visual field. In this way, the visual evoked potential signal carries some of the properties of the stimuli on which the user concentrates, such as the frequency and phase of the signal. Accordingly, it is possible to tell which stimuli the user is gazing at by comparing the extracted signal with the stimuli and then convert such information into computer instructions (Zhao et al., 2017).

According to a previous report (Regan, 1989), the low (4–12 Hz), medium (12–30 Hz) and high frequency range (−30 Hz) are the three frequency ranges to elicit an SSVEP. In general, the low frequency range could elicit larger amplitude SSVEP responses than the medium and high frequency ranges. Moreover, the larger the amplitude of the SSVEP, the easier its detection. Thus, many studies used low and medium frequency ranges to elicit SSVEP (No-Sang et al., 2015). However, low and medium frequency SSVEP ranges interfere with the alpha rhythm, and could cause an epileptic seizure (Fisher et al., 2005). The weakest SSVEP is found in the high frequency range (Diez et al., 2013). However, high frequency stimulation has the advantage of greatly decreasing the visual fatigue, caused by flickering, so that these stimuli can be used to develop a more comfortable SSVEP-based brain–computer interface (BCI) (Molina and Mihajlovic, 2010; Volosyak et al., 2011; Diez et al., 2013, 2014). However, the frequencies lower than 4 Hz seemed to be abandoned in eliciting SSVEP.

Usually, the traditional stimulus mode of SSVEP is light flashing or graph flipping (Lin et al., 2006), using a flickering light (the change of brightness) as a stimulus paradigm. The paradigm based on a flickering light is likely to cause visual fatigue and discomfort with a consequent decrease of the recognition accuracy. In recent years, BCI paradigms based on motion perception have been proposed (Snowden and Freeman, 2004). Xie et al. (2012) designed a BCI paradigm using Newton’s rings based on steady-state motion visual evoked potential (SSMVEP) that increased the recognition accuracy to a favorable level (Xie et al., 2014). Yan et al. (2018) designed four novel stimulus paradigms based on basic motion modes and demonstrated that any stimulus paradigms with periodic motion can induce SSMVEPs. However, all these research studies on the SSMVEP used the low and medium frequency bands. In fact, many human-related motion frequencies in our daily lives are lower than 1 Hz, such as the stride motion frequency, wave frequency, etc. In particular, the stride motion frequency refers to the number of times the legs alternate within 1 s. Human’s normal walking speed ranges from 10 to 80 steps per minute, which means that the stride motion frequency ranges from 0.08 to 0.67 Hz. The stride motion frequencies are lower than 4 Hz. Accordingly, few studies have reported the recognition of stride motion frequencies based on SSVEP. Moreover, all the SSVEP paradigms are based on flicker or motion separately. There is no paradigm combining flickering light and motion. Also, the characteristics of the brain response in the motion paradigm modulated by the change of brightness are unclear.

Besides, many researchers used SSVEP to control external moving devices, such as a wheelchair (Jzau-Sheng et al., 2014), a lower limb exoskeleton (No-Sang et al., 2015), a hand orthosis (Ortner et al., 2011), etc. However, all the stimulations were flickering LEDs or squares in the screen. The meanings of the targets were designed by the experimenters. Subjects would not know the meaning of the stimulation if the experimenter did not tell them the meaning. Consequently, it could be better for subjects to understand the meaning of the paradigms if the paradigms had similarity with the controlled devices.

In this study, to classify the different stride motion frequencies from the visual area in brain and to explore the characteristics of the brain response to the motion paradigm modulated by the change of brightness, the light spot humanoid motion paradigm is proposed for the first time. The paradigm was modulated with high-frequency flicker and low-frequency motion to produce humanoid walking. The flicker frequency was higher than 30 Hz so that the human eyes felt almost no flicker. Additionally, the motion frequency was the human stride frequency. The characteristics of the brain response to this paradigm were examined. In addition, canonical correlation analysis (CCA) was used to distinguish the different stride frequencies online. Then, the accuracies of distinguishing the different stride frequencies with the same and different high frequencies were compared. Finally, based on our previous rehabilitation training robot (Zhang et al., 2015), we employed the designed paradigm to control a lower limb robot with the same frequency stimulation. The robot provided motor feedback according to the identification of the electroencephalograph (EEG) signals when subjects stared at the humanoid motion paradigms with different stride frequencies. Moreover, the angles of the knee joint of the robot were obtained to test whether the feedback motion frequency was the same as the visual stimulation frequency.

## Materials and Methods

### Ethics Statement

Ten healthy male subjects (ages 23–27) participated in the experiments. They were all volunteers from Xi’an Jiaotong University. Subjects were studied after giving informed written consent in accordance with a protocol approved by the institutional review board of Xi’an Jiaotong University.

### Design of the Light Spot Humanoid Motion Paradigm Modulated by the Change of Brightness

**FIGURE 1:**
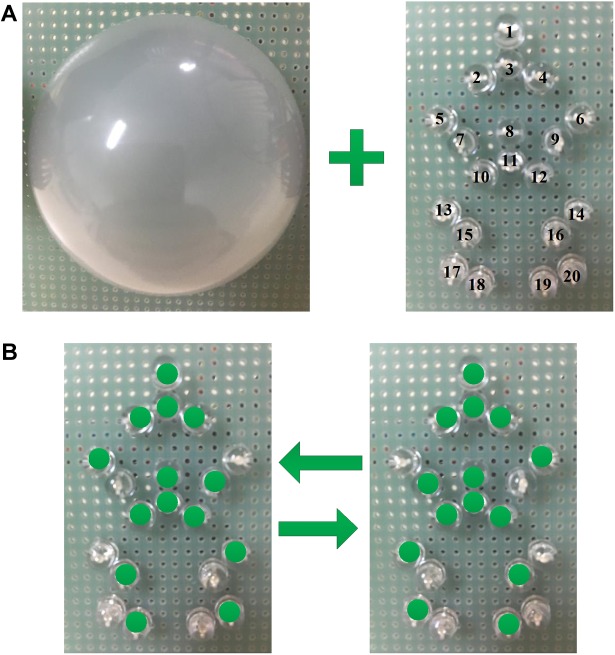
The light spot humanoid motion stimulus paradigm modulated by the change of brightness. **(A)** Are 20 green straw-hat LEDs and a hemispherical lampshade, and **(B)** is the moving process of the light spot human.

In this study, 20 green straw-hat LEDs were chosen as the visual stimulation which is shown in **Figure 1**. The 20 LEDs were placed in a human form based on the main points of the human skeleton, which included head, shoulder, elbow, spine, hip, knee, and ankle. Those LEDs were divided into three groups. Group 1 included the points of the human skeleton that almost immovable in human walking and comprised LED 1, LED 2, LED 3, LED 4, LED 8, LED 10, LED 11, and LED 12, which showed head, shoulder, spine, and hip. Thus, LEDs in group 1 were designed for the high-frequency flicker. Group 2 comprised LED 5, LED 9, LED 14, LED 15, LED 18, and LED 20, which showed arms and legs in stance phase. Group 3 comprised LED 6, LED 7, LED 13, LED 16, LED 17, and LED 19, which showed arms, and legs in swing phase. The LEDs in group 2 and group 3 flickered alternately at low frequency, which can form the walking motion as shown in **Figure 1B**. The motion was similar to the motion when the human traffic light turned green.

In addition, to create a more effective sense of motion, a lampshade was used to cover the LEDs. The hemispherical lampshade was made of light diffusing material with 85% light transmittance and each LED projected its light to the lampshade. The point light source became the surface light source. The light spot humanoid motion with a lampshade was more like the motion displayed on a screen. On the other hand, the lampshade made the paradigm more pedestrian and avoided the visual discomfort of the subjects due to the stimulation of the LEDs. Also, subjects were not able to see those LEDs that had been turned off in the designed paradigm with a lampshade. And those LEDs that had been turned off might confuse the subjects to recognize the shape of the paradigm.

Additionally, the STM32F103RB (ST-Microelectronics company, Geneva, Switzerland) was chosen as the microcontroller to control the flickering of the LEDs. By using the timer of STM32, a pulse width modulation (PWM) signal can be produced. Changing the duty ratio of the PWM signal could change the brightness of the LEDs. The duty ratios of the PWM to light up LEDs in group 1, group 2, and group 3 were changed as *DR*1, *DR*2, and *DR*3, respectively. The sum of *DR*1, *DR*2, and *DR*3 was the whole brightness of the paradigm. And the overall brightness was changed at frequency *F*. At the same time, *DR*2 and *DR*3 had a phase difference of π, and the motion was achieved by this phase difference. In addition, another timer was used to guarantee the accuracy of the cycle. The time interval was 1 ms.

(1) DR1=(300+300×sin(2×π×F×t)/900)

(2) DR2=(250+200×Sin(2×π×F×t)+200×Sin(2×π×f×t))/900

(3) DR3=(250−200×sin(2×π×F×t)−200×sin(2×π×f×t))/900

where *F* denotes the flicker frequency, *f* represents the motion frequency, and *t* is the time interval.

### EEG Signal Measurement

The EEG signals were recorded from six EEG electrodes connected to a g.GAMMAbox and a g.USBamp EEG amplifier (g.tec Guger Technologies OG., Graz, Austria). The brain electrophysiological responses induced by visual stimulation were mainly distributed in the occipital region. So the six EEG electrodes were placed at PO3, PO4, POz, O1, O2, and Oz based on the international 10–20 system. The unilateral (left or right) earlobe was used as the recording reference and the Fpz was used as ground. All electrodes’ impedances were kept below 5 k Ohm during the experiments. The sampling frequency was 1200 Hz.

### Lower Limb Robot

The XYKXZFK-9 lower limb robot (Xiangyu Medical Equipment Co. Ltd., China) was chosen to obtain motor feedback. This robot had been described in our previous research (Zhang et al., 2015). The robot was able to drive the lower extremity of users with reciprocating exercise similar to treadmill exercise. The knee joint was driven by the motor and other joints were follow-up. The control instructions of the robot can be sent through serial communication with computer. The control instructions included the speed of the pace, amplitude of the pace, etc. In addition, the angles of the knee joint during the movement can be obtained from the encoder.

### Experimental Design

Two offline experiments described in **Table 1** were designed to explore the characteristics of the brain response to the light spot humanoid motion paradigm modulated by the change of brightness. In addition, one online experiment described in **Table 1** was designed to demonstrate the feasibility of recognizing stride motion frequencies and the EEG-driven lower limb robot with the same frequency stimulation.

**Table 1:** Experimental design.

	Mode	Frequency (Hz)	Duration (s)	Time interval (s)	Total number of cycles for each target
*E1*	High frequency (*F*)	35, 45, 55, 65, 75, 85	5	5	10
*E2*	L & M modulated (*F*, *f*)	(45, 0.2), (45, 0.4), (45, 0.6), (43, 0.2), (43, 0.4), (43, 0.6), (41, 0.2), (41, 0.4), (41, 0.6).	5	5	20
*E3*	L & M modulated (*F*, *f*)	M1: (41, 0.2), (41, 0.4), (41, 0.6). M2: (41, 0.2), (45, 0.4), (43, 0.6).	1, 2, 3, 4, 5	12	20

In the offline experiments (E1 and E2), the subjects were asked to sit on a comfortable chair in a quiet and ordinarily lit office room, and the designed paradigm was placed 40 cm in front of them. The LEDs were flickered in the way described in Section “Design of the Light Spot Humanoid Motion Paradigm Modulated by the Change of Brightness” for 5 s and turned off for 5 s automatically. All the subjects were required to stare at the paradigm for 5 s per trial with an interval of 5 s. There were 10 trials for each high frequency in E1, the high frequencies were 35, 45, 55, 65, 75, and 85 Hz, successively. Only the LEDs in group1 were lightened during the experiment E1. In addition, there were 20 trials for each stimulus frequency in E2. Specifically, 0.2, 0.4, and 0.6 Hz were chosen as the motion frequencies, while 45, 43, and 41 Hz were chosen as the flicker frequencies. Thus, the stimulus frequencies were: (45 Hz, 0.2 Hz), (45 Hz, 0.4 Hz), (45 Hz, 0.6 Hz), (43 Hz, 0.2 Hz), (43 Hz, 0.4 Hz), (43 Hz, 0.6 Hz), (41 Hz, 0.2 Hz), (41 Hz, 0.4 Hz), and (41 Hz, 0.6 Hz). As a result, the offline experiments E2 consisted of nine rounds. Throughout all the experiments, the EEG signals and the time were automatically recorded in the computer hard disk. After the experiments, the subjects were asked about the intuitive perception of the designed paradigms and the meaning of the paradigms.

Based on the offline experimental results, we explored the characteristics of the brain response to the light spot humanoid motion paradigm. Then, we chose the optimal frequency as the high-flicker frequency to perform the online experiments.

In the online experiments (E3), the subjects were fixed on the lower limb robot (XYKXZFK-9). And the standing angle of the robot was 70°. The three designed paradigms were placed 80 cm in front of the subjects. The motion frequencies were 0.2, 0.4, and 0.6 Hz, respectively. The LEDs flickered in the same way described in Section “Design of the Light Spot Humanoid Motion Paradigm Modulated by the Change of Brightness” for 5 s. Then, the LEDs were turned off for 12 s while the lower limb robot drove the subjects to perform lower limb movements with different stride frequencies according to the results of the identification. Additionally, the focused target was identified by CCA. Subjects were required to stare at the three targets one by one, there were 20 trials for each target. The stimulation duration was 4, 3, 2, and 1 s in succession. The process of the experiments was the same as described above. Notably, two different methods were used to present the humanoid motion with three different motion frequencies. One was that the high flicker frequency was the same (HFS). For example, the stimulus frequencies were (41 Hz, 0.2 Hz), (41 Hz, 0.4 Hz), and (41 Hz, 0.6 Hz). The other one was that the high flicker frequency was different (HFD). For example, the stimulus frequencies were (41 Hz, 0.2 Hz), (45 Hz, 0.4 Hz), and (43 Hz, 0.6 Hz). During the experiments, the EEG signals and the angles of the knee joint of the lower limb robot were automatically recorded in the computer hard disk.

### Pre-processing of the EEG Data

The data collected from each subject were analyzed independently. The corresponding EEG data segments were extracted according to the starting time and ending time of the stimulus. The notch filtering from 48 to 52 Hz was conducted to remove the power line interface and the band pass filtering from 30 to 95 Hz was performed to remove the low frequency drift.

### Common Average Reference

Common average reference (CAR) is commonly used in EEG, where it is necessary to identify small signal sources in very noisy recordings. The CAR is calculated as described in Equation (4). The mean value of all the electrodes is removed for a certain electrode (Alhaddad, 2012).

(4) $$O_{z}=O_{Z}-(O_{1}+O_{2}+{\mathrm{PO}}_{3}+{\mathrm{PO}}_{4}+{\mathrm{PO}}_{z}+O_{z})/6$$

### Signal-to-Noise Ratio

To evaluate the significance of the amplitude frequency response in the EEG data, the signal-to-noise ratio (SNR) of the SSVEP is computed. The SNR of the SSVEP is calculated as the ratio between the power of a given frequency and the average power of its 20 surrounding neighbors.

(5) $$\mathrm{SNR}(f_{n})=P(f_{n})/\left(\frac{1}{20}\times \sum_{\begin{array}{l} \text{q=-10}\\ \text{ q}\neq \text{0} \end{array}}^{10}P(f_{n+q})\right)$$

where *P*(*f_n_*) represents the Fourier power of a given frequency *f_n_*.

Additionally, the EEG data epochs were extracted every 5 s. So the spectral resolution was 0.2 Hz.

### Canonical Correlation Analysis

Canonical correlation analysis is widely used in SSVEP target recognition, where it is used to calculate the correlations between reference signals and multi-channel EEG data (Lin et al., 2006). The formula to calculate the correlation coefficient is as described in Equation (6).

(6) $$\rho =\underset{w_{x},w_{y}}{\max}\frac{E[w_{x}^{T}{\mathrm{XY}}^{T}w_{y}]}{\sqrt{E[w_{x}^{T}{\mathrm{XX}}^{T}w_{y}]E[w_{x}^{T}{\mathrm{YY}}^{T}w_{y}]}}$$

where *X* is the reference signals and *Y* is the EEG data.

In this study, the reference signals *X* were composed of eight groups of sine and cosine signals which were described as follows.

(7) $$X=\left\{\begin{array}{l} \sin(2\times \pi \times (F-f)\times t)\\ \cos(2\times \pi \times (F-f)\times t)\\ \sin(2\times \pi \times F\times t)\\ \cos(2\times \pi \times F\times t)\\ \sin(4\times \pi \times F\times t)\\ \cos(4\times \pi \times F\times t)\\ \sin(2\times \pi \times (F+f)\times t)\\ \cos(2\times \pi \times (F+f)\times t) \end{array}\right\},\;t=\frac{1}{\mathrm{Fs}},\;\frac{2}{\mathrm{Fs}},\;\ldots ,\;\frac{T}{\mathrm{Fs}}$$

Where *F* is the high flicker frequency and *f* is the low motion frequency.

After CCA is performed separately on each stimulus, the target can be determined by the maximum canonical coefficient.

### Statistical Analysis

Statistical analysis was conducted using Wilcoxon signed-rank test which is a non-parametric statistical hypothesis test. Statistical significance is defined as *p* < 0.05.

## Results

### Offline Experimental Results of the Brain Response to High Frequency Stimulation

**FIGURE 2:**
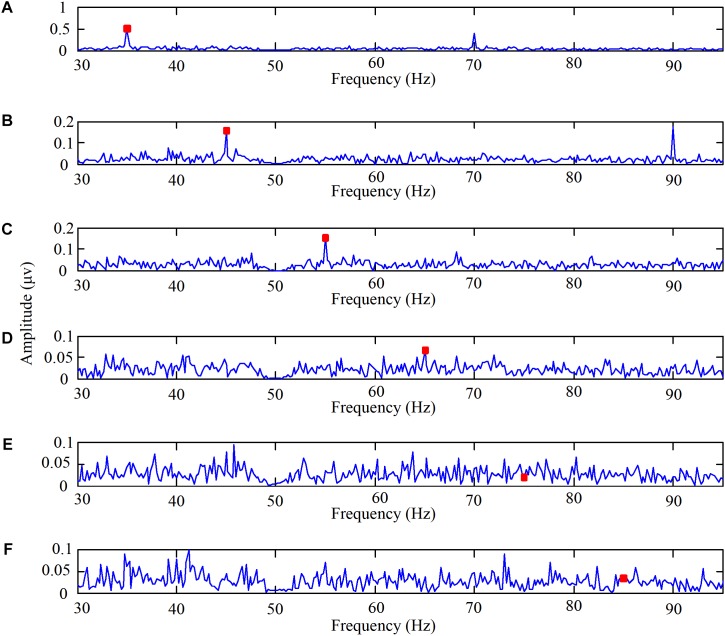
The EEG spectra when one subject stared at the high frequency flicker stimulations. **(A)** The high flicker frequency is 35 Hz, **(B)** the high flicker frequency is 45 Hz, **(C)** the high flicker frequency is 55 Hz, **(D)** the high flicker frequency is 65 Hz, **(E)** the high flicker frequency is 75 Hz, and **(F)** the high flicker frequency is 85 Hz.

The experiments on the brain response to high frequency stimulation were designed to examine the characteristics of the brain response to high frequency stimulation and to determine the appropriate frequency for use as the high flicker frequency in our designed paradigm. The EEG spectra of one subject staring at the high-frequency flicker stimulations are shown in **Figure 2**. The high frequencies were 35, 45, 55, 65, 75, and 85 Hz, respectively. Before performing the fast Fourier transform, the EEG data of ten cycles corresponding to each frequency were superimposed and averaged on the time domain. Additionally, six channel signals were fused to a single channel data according to CAR. The data shown in **Figure 2** reveals that when the stimulation frequency was less than 65 Hz the spectrum had a significant peak at the stimulation frequency, but when the stimulation frequency was 75 or 85 Hz, the amplitude at the stimulation frequency in the spectrum was not significant.

Interestingly, the amplitude at the frequency in the spectrum was significant even when the second harmonic frequency was higher than 65 Hz. Taking the stimulation frequency of 45 Hz as an example, the second harmonic frequency was 90 Hz. The data shown in **Figure 2B** reveals that the peak in the spectrum at 90 Hz was significant. Even though the mechanism was not clear, we can add the second harmonic frequency as another feature to perform online identification.

**FIGURE 3:**
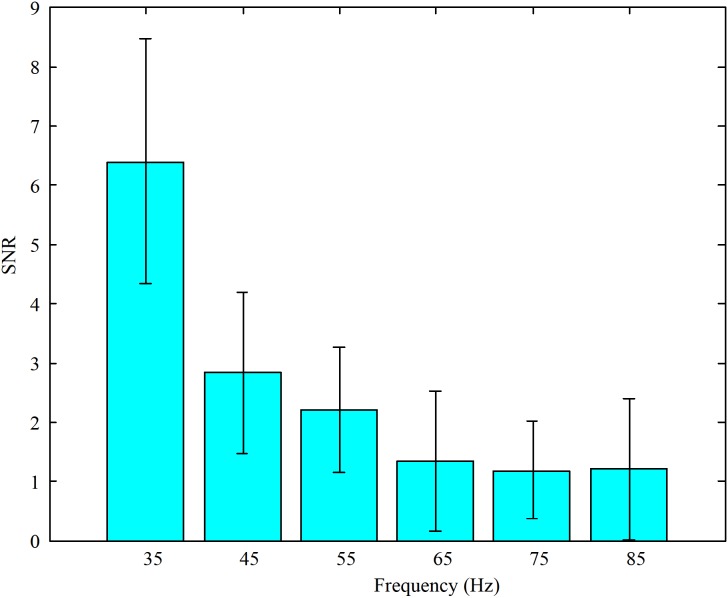
The average SNRs of EEG data when subjects stared at the high frequency stimulations (35, 45, 55, 65, 75, and 85 Hz, respectively).

Furthermore, in order to select the appropriate high flicker frequency for our designed paradigm, the SNRs were calculated. The average SNRs of the EEG data when subjects stared at the high frequency stimulations are shown in **Figure 3**. These results illustrate that the SNRs decreased as the stimulus frequencies increased. According to the feedback from the subjects, all the subjects felt the flickering of the LEDs when the frequency was 35 Hz and felt almost no flickering of the LEDs at other frequencies. Accordingly, the motion paradigm modulated by the change of brightness was designed without the sense of flicker. So the frequencies around 45 Hz were chosen as the high flicker frequencies in our designed paradigm.

### Offline Experimental Results on the Brain Response to the Light Spot Humanoid Motion Stimulation

**FIGURE 4:**
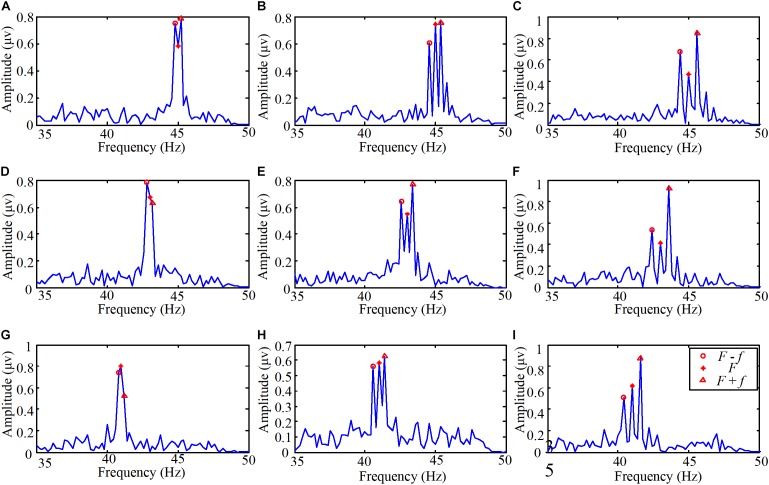
The EEG spectra when one subject stared at the light spot humanoid motion stimulations. **(A)** The flicker frequency *F* was 45 Hz and the motion frequency *f* was 0.2 Hz, **(B)** the flicker frequency *F* was 45 Hz and the motion frequency *f* was 0.4 Hz, **(C)** the flicker frequency *F* was 45 Hz and the motion frequency *f* was 0.6 Hz, **(D)** the flicker frequency *F* was 43 Hz and the motion frequency *f* was 0.2 Hz, **(E)** the flicker frequency *F* was 43 Hz and the motion frequency *f* was 0.4 Hz, **(F)** the flicker frequency *F* was 43 Hz and the motion frequency *f* was 0.6 Hz, **(G)** the flicker frequency *F* was 41 Hz and the motion frequency *f* was 0.2 Hz, **(H)** the flicker frequency *F* was 41 Hz and the motion frequency *f* was 0.4 Hz, **(I)** the flicker frequency *F* was 41 Hz and the motion frequency *f* was 0.6 Hz.

The experiment on the brain response to the light spot humanoid motion stimulation was designed to explore the characteristics of the brain response to the motion modulated by the change of brightness. The EEG spectra of one subject staring at the various stimulations are shown in **Figure 4**. The flicker frequencies and motion frequencies (*F, f*) in the stimulations were (45 Hz, 0.2 Hz), (45 Hz, 0.4 Hz), (45 Hz, 0.6 Hz), (43 Hz, 0.2 Hz), (43 Hz, 0.4 Hz), (43 Hz, 0.6 Hz), (41 Hz, 0.2 Hz), (41 Hz, 0.4 Hz), and (41 Hz, 0.6 Hz) respectively.

The process of the EEG data was the same as the experiment on the brain response to high frequency stimulation (see details in Section “Pre-processing of the EEG Data”). Then the EEG data were filtered with a CCA-based filter. The duration of the stimulation was 5 s. So the spectral resolution was 0.2 Hz. As shown in **Figure 4**, peaks in amplitude can be precisely identified at the high flicker frequency, the sums and differences of the flickering frequency and motion frequency. In other words, the peaks were mainly evoked at the frequencies *F*, *F* + *f*, and *F* – *f*. Therefore, even the frequency which is lower than 1 Hz does not easily induce SSVEP, humanoid motion modulated with high-frequency light intensity scintillation can induce regular features. Moreover, from the inquiries after the experiments, we determined that all the subjects could hardly aware of the modulation with high-frequency flicker.

**FIGURE 5:**
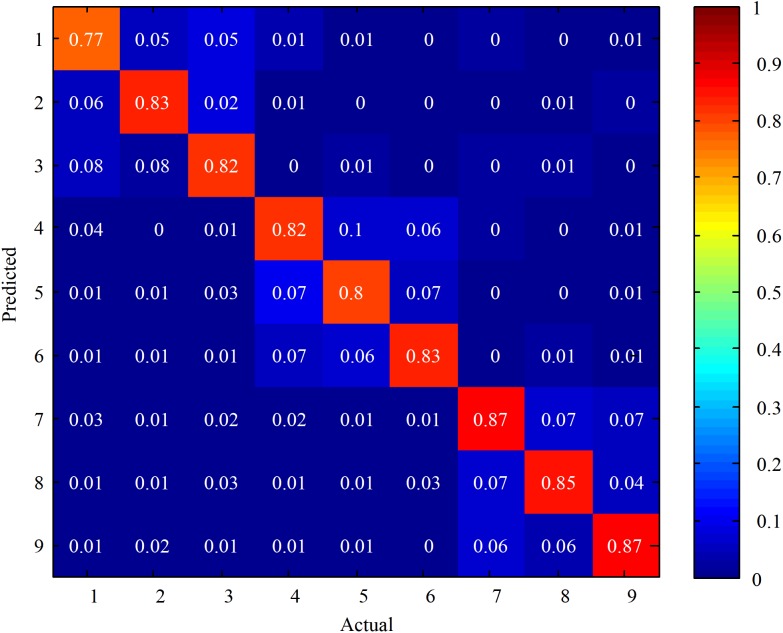
Confusion matrices for the stimulations at 5 s stimulus time among all the subjects. The color scale reveals the classification accuracies, the diagonals labeled with the correct classification accuracies.

Then, based on the above described results, Equation (7) was chosen as the reference signal to perform the CCA with the EEG data. Additionally, the offline classification accuracies were calculated among all the ten subjects. In order to compare the relative classification performance of each target, **Figure 5** shows confusion matrices for the stimulations for 5 s among all the subjects. The label ‘1’ to ‘9’ referred to the stimulations with the following stimulus frequencies (45 Hz, 0.2 Hz), (45 Hz, 0.4 Hz), (45 Hz, 0.6 Hz), (43 Hz, 0.2 Hz), (43 Hz, 0.4 Hz), (43 Hz, 0.6 Hz), (41 Hz, 0.2 Hz), (41 Hz, 0.4 Hz), and (41 Hz, 0.6 Hz), respectively. It was observed that the main misclassification occurred at those stimulations with the same flickering frequency. Also, the lower the flicker frequency of the stimulation, the higher the classification accuracy.

### Online Experimental Results

In the online experiments, CCA was used to calculate the accuracies of classifying three different motion frequencies. The online classification accuracies of the three stimulations by two different methods to depict the humanoid motion are shown in **Table 2**. The stimulus duration was 5 s, and the average accuracies by the two methods reached 87 and 95%, respectively. Remarkably, the classification accuracies of six subjects reached 100% with the designed paradigm with the different high flicker frequencies. In addition, the accuracies of most subjects were high, but subject 6 and 9 exhibited low classification accuracies. The low accuracies implied that the value of the canonical coefficient was low. The designed paradigm did not induce very strong SSVEP at the desired frequencies in these two subjects.

**Table 2:** Online classification accuracies with 5 s stimulus duration.

	Accuracy (high frequencies keep in the same)	Accuracy (high frequencies are different)
S1	0.93	1
S2	0.91	1
S3	0.97	1
S4	0.94	1
S5	0.95	0.97
S6	0.74	0.8
S7	0.97	1
S8	0.84	1
S9	0.62	0.85
S10	0.87	0.92
Average	0.87 ± 0.11	0.95 ± 0.07

**FIGURE 6:**
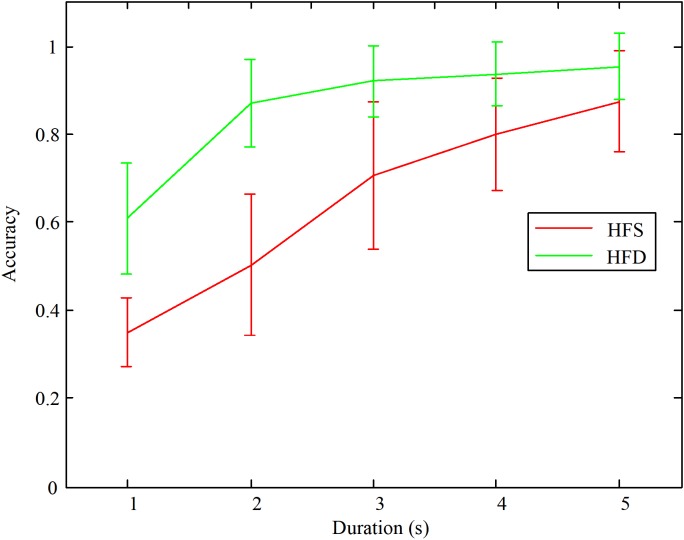
The average classification accuracies among all the subjects with different stimulation durations.

Subsequently, the accuracies were averaged among all the subjects. The online accuracies with different stimulus durations are shown in **Figure 6**. We test normality and homogeneity of variances between the accuracies of distinguishing the different stride frequencies with the same and different high frequencies. We found that the distributions of the accuracies in 5 s duration using HFD was not normal (*p* = 0.002 < 0.05, Kolmogorov–Smirnov test). The distributions of accuracies in other groups were normal distribution. Thus, we chose Wilcoxon signed-rank test, which had no assumption on normality, to compare the accuracies of distinguishing the different stride frequencies with the same and different high frequencies. Statistical significance is defined as *p* < 0.05. The results showed that the classification accuracies of the designed paradigm using HFS were significantly lower than the accuracies of the designed paradigm using HFD when the durations were 5, 4, 3, 2, and 1 s [(*z* = −2.807, *p* = 0.005), (*z* = 2.710, *p* = 0.007), (*z* = −2.805, *p* = 0.005), (*z* = −2.805, *p* = 0.005), (*z* = −2.807, *p* = 0.005) respectively]. In addition, the classification accuracies of both methods were decreasing as the duration decreased. Specifically, when the stimulation duration was less than 3 s, there was a tendency for a sharp decrease in the classification accuracies.

**FIGURE 7:**
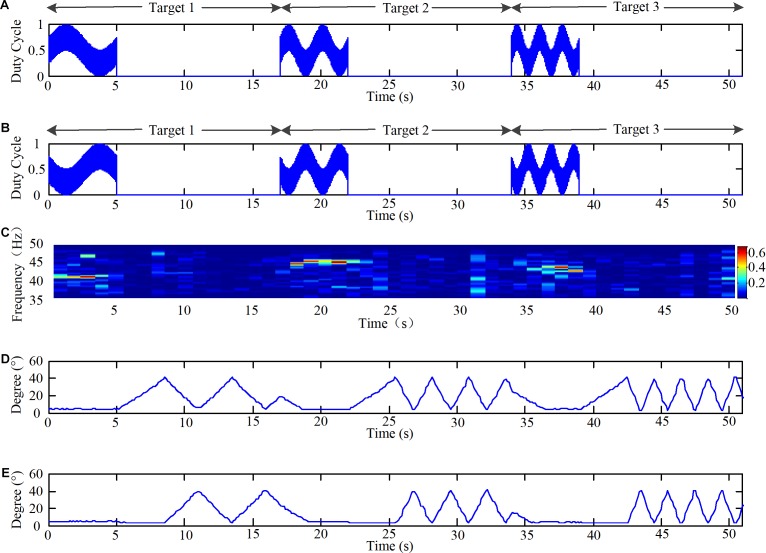
Time domain of duty ratio of the PWM, time-frequency map of EEG data, and angles of knee joint of the lower limb robot. **(A)** The time domain of duty ratio of the PWM of LEDs in group 2 in three targets. **(B)** The time domain of duty ratio of the PWM of LEDs in group 3 in three targets. **(C)** The time-frequency map of EEG data during the online experiment and the color scale reveals the values of power spectrum. **(D)** The angles of left knee joint of the lower limb robot during the online experiment. **(E)** The angles of right knee joint of the lower limb robot during the experiment.

Finally, the angles of the knee joint of the lower limb robot were acquired at the same time during the acquisition of the EEG signals during the online experiments. The time domain of the duty ratio of the PWM, time-frequency map of the EEG data, and angles of the knee joint of the lower limb robot are shown in **Figure 7** for one subject who successively stared at three targets (M2: (41 Hz, 0.2 Hz), (45 Hz, 0.4 Hz), (43 Hz, 0.6 Hz)).

The paradigms would light on for 5 s in the way described in Section “Design of the Light Spot Humanoid Motion Paradigm Modulated by the Change of Brightness,” and light out for 12 s, as shown in **Figures 7A,B**. Thus, the EEG data for the 0–5 s, 17–22 s, and 34–39 s periods were the data processed online. The EEG data were filtered with a CCA-based filter. Then, the time-frequency map was generated as shown in **Figure 7C**. The mapre 7C. The map revealed that the stimulations induced corresponding frequencies in the brain. Additionally, the system successfully recognized the intentions of the subjects as they stared at the three targets successively. It should be noted that our robot moved the left leg first in every operation and the speed of the first step is a fixed value in **Figure 7D**. Furthermore, when the subject stared at target 1 when the motion frequency of the stimulus was 0.2 Hz, as shown in **Figures 7A,B**, from 0 to 5 s, the robot’s movement frequency was also 0.2 Hz, as shown in **Figures 7D,E** from 10 to 15 s. Thus, these results demonstrated that a brain-controlled lower-limb robot with the same stimulus frequency and actual movement frequency was feasible.

## Discussion and Conclusion

In this study, a light spot humanoid motion paradigm modulated by the change of brightness was proposed. In addition, the characteristics of the brain response to the motion paradigm modulated by the change of brightness were determined for the first time. By using these characteristics, we realized the recognition of the stride motion frequencies which were not in the regular frequency bands of the SSVEP. Moreover, we used the designed paradigm to control a lower limb robot by EEG with the same frequency stimulation.

The designed paradigm was created by the modulation of the motion and the change of brightness. It was an imitation of the liquid cry crystal display (LCD) monitor which is a usual way to elicit an SSVEP. The refresh rate of the designed paradigm was the high flicker frequencies (45, 43, and 41 Hz), while the screen refresh rate of the LCD was 60 Hz or larger. However, 60 Hz was too high to evoke available SSVEP. Thus, it can be speculated that if the stride motion was displayed on an LCD monitor with a lower screen refresh rate (lower than 45 Hz), the modulation phenomena in the EEG spectra also could be occurred. Furthermore, one group’s LED in the designed paradigm flickered in the traditional way. Subjects did not feel the flicker of the paradigm using the high flicker frequency, while the other two groups’ LEDs had a phase difference of π to produce alternate changes. In addition, these LEDs were covered with a lampshade which made the alternate changes generate a motion. And that was just like a motion displayed on a screen instead of an individual flicker. The total brightness of the paradigm was *DR*1 + *DR*2 + *DR*3 = *DR*1 + 500, and there was no motion frequency in *DR*1. Accordingly, the motion frequencies did not reflect in the total brightness.

Moreover, only stimulation frequencies within a specific frequency range can evoke strong SSVEP (Vialatte et al., 2010). Thus, some researchers used multiple frequencies to encode more targets (Teng et al., 2010; Zhang et al., 2012). Earlier, Mukesh et al. (2006) proposed a novel dual-frequency stimulation method to increase the number of selections in BCI. The peaks in the spectrum mainly occurred at *F*1, *F*2, *F*1 + *F*2, 2 × *F*2, *F*1 + 2 × *F*2, 2 × *F*1 + 2 × *F*2, 3 × *F*2, *F*1 + 3 × *F*2, 2 × *F*1 + 3 × *F*2. Also, Shyu et al. (2010) obtained a dual-frequency stimulation with two flickering LEDs. The peaks mainly occurred at *F*1, *F*2, 2 × *F*2 – *F*1, 2 × *F*1 – *F*2. More recently, Chang et al. (2014) proposed an amplitude-modulated visual stimulation, and the peaks occurred at 2 × *f_c_*, 2 × *f_m_*, *f_c_* ± *f_m_*, *f_c_* ± 3 × *f_m_*, 2 × *f_c_* ± 4 × *f_m_*. All the above-mentioned studies modulated the frequencies based on brightness and the results were not exactly the same. However, to the best of our knowledge, no study has reported the characteristics of the brain response to the motion paradigm modulated by the change of brightness. In this study, the high-frequency flicker and stride motion were modulated by the visual stimulation. The results of this study showed that the designed paradigm induced the main frequencies at *F*, *F* - *f*, and *F* + *f* (*F*: flicker frequency, *f*: motion frequency) in the brain. The motion frequency was modulated onto the side band of the high flicker frequency. However, there were no peaks at other modulation frequencies (such as *F* ± 2 × *f*) in the spectrum, which was different from the existing research. The reasons why those combined frequencies occurred are still unknown and need to be explored in the future. Besides, the frequency to elicit SSVEP was larger than 4 Hz according to (Regan, 1989). All these studies used the frequencies in the regular frequency regions (larger than 4 Hz), and no study considered how frequencies lower than 4 Hz could be used. In this study, we used the motion frequencies lower than 1 Hz, and demonstrated that such frequencies (lower than 1 Hz) can be used by modulated them with high flicker frequencies.

In addition, based on the online experimental results, the classification accuracies of the designed paradigm using HFD were significantly higher than the accuracies of the designed paradigm using HFS. The reason was that the paradigm using HFD had one more feature, namely the high flicker frequencies, than the paradigm using HFS. This finding indicated that not only the motion frequency *f* was different, but also the flicker frequency *F* in the reference signal was different when CCA was used to perform the classification. Regarding the visual perception, the subjects hardly felt the difference between the two methods. However, the difference can be detected through the EEG data. Moreover, the average accuracy reached 95% with the designed paradigm using HFD, and the accuracy was more than enough for the application in the BCI systems.

Finally, based on the EEG-driven lower limb rehabilitation training system, which we proposed in our previous study (Zhang et al., 2015), we applied the designed paradigm to control a robot by EEG with the same frequency stimulation. Even though in our previous study (Zhang et al., 2015) the virtual reality was designed to provide visual feedback, the stimulus was the normal SSMVEP paradigm. Accordingly, subjects could not know the meaning of the paradigm if the experimenter did not tell them. In addition, other SSVEP-based exoskeleton robots (Kwak et al., 2014) had used normal light scintillation stimulation. Also, there was no similarity between the visual stimulus and the motor feedback. In this study the humanoid motion paradigm was designed to induce SSVEP to control a robot. Additionally, all the subjects were asked what the paradigm was. And they responded that the paradigm was a walking human and the walking speeds were different. Thus, the designed paradigm has similarity with the motor feedback.

Overall, this study mainly determined the characteristics of the brain response to the motion paradigm modulated by the change of brightness and distinguished different stride motion frequencies from visual areas of the brain by designing a novel SSVEP paradigm. We found that the peaks were mainly evoked at the frequencies *F*, *F* + *f*, and *F* – *f* in the spectrum of the EEG data as the brain response to the paradigm. In addition, based on the findings, we achieved the recognition of the different stride motion frequencies by modulating the change of brightness on motion. Finally, the online experiment demonstrated the feasibility of the same frequency stimulation for an EEG-driven robot. In our future studies, we plan to increase the number of LEDs and divide more groups in the paradigm to make the paradigm more like a human with more realistic movement. And we will implement other algorithms to process the EEG data to improve the performance. Besides, all the subjects in the experiments are males. That may limit the extensibility of the results to general population. Thus, we may try to recruit female subjects to explore the sex effects. More importantly, visual stimulation can stimulate the motor cortex through the mirror neurons (Rizzolatti and Craighero, 2004) in the human brain. Nojima et al. (2012) also found that the mirror visual feedback has an important role in brain plasticity in the motor cortex. Thus an important question is whether it is feasible to use the SSVEP in rehabilitation training if the humanoid motion paradigm was chosen as the stimulation. Finding such an answer will be an interesting follow-up study.

## Data Availability Statement

The datasets [the main data about the paradigm modulated by the change of brightness and motion] for this study can be found in the [FigShare] https://figshare.com/s/094b2fa683acca657991.

## Author Contributions

XinZ: study design, manuscript preparation, data acquisition, and analysis. GX: study design and study supervision. XunZ: helped with the experiments. QW: helped in polishing the paper.

## Conflict of Interest Statement

The authors declare that the research was conducted in the absence of any commercial or financial relationships that could be construed as a potential conflict of interest.
